# Modeling trophic dependencies and exchanges among insects’ bacterial symbionts in a host-simulated environment

**DOI:** 10.1186/s12864-018-4786-7

**Published:** 2018-05-25

**Authors:** Itai Opatovsky, Diego Santos-Garcia, Zhepu Ruan, Tamar Lahav, Shany Ofaim, Laurence Mouton, Valérie Barbe, Jiandong Jiang, Einat Zchori-Fein, Shiri Freilich

**Affiliations:** 1 Newe Ya’ar Research Center, The Agricultural Research Organization, Ramat Yishay, Israel; 2 Agricultural Research and Development Center, Southern Branch (Besor), Israel; 3 Department of Entomology, Hebrew University of Jerusalem, Rehovot, Israel; 4 Department of Microbiology, College of Life Sciences, Nanjing Agricultural University, Nanjing, 210095 China; 5 CNRS, Laboratoire de Biométrie et Biologie Evolutive UMR CNRS 5558, Université de Lyon, Université Claude Bernard, F-69622 Villeurbanne, France; 6 Institut de biologie François-Jacob, GenoscopeCEA, Genoscope, Evry, France

**Keywords:** *Bemisia tabaci*, Metabolic interactions, Network analysis, Symbionts

## Abstract

**Background:**

Individual organisms are linked to their communities and ecosystems via metabolic activities. Metabolic exchanges and co-dependencies have long been suggested to have a pivotal role in determining community structure. In phloem-feeding insects such metabolic interactions with bacteria enable complementation of their deprived nutrition. The phloem-feeding whitefly *Bemisia tabaci* (Hemiptera: Aleyrodidae) harbors an obligatory symbiotic bacterium, as well as varying combinations of facultative symbionts. This well-defined bacterial community in *B. tabaci* serves here as a case study for a comprehensive and systematic survey of metabolic interactions within the bacterial community and their associations with documented occurrences of bacterial combinations. We first reconstructed the metabolic networks of five common *B. tabaci* symbionts genera (*Portiera*, *Rickettsia*, *Hamiltonella*, *Cardinium* and *Wolbachia*), and then used network analysis approaches to predict: (1) species-specific metabolic capacities in a simulated bacteriocyte-like environment; (2) metabolic capacities of the corresponding species’ combinations, and (3) dependencies of each species on different media components.

**Results:**

The predictions for metabolic capacities of the symbionts in the host environment were in general agreement with previously reported genome analyses, each focused on the single-species level. The analysis suggests several previously un-reported routes for complementary interactions and estimated the dependency of each symbiont in specific host metabolites. No clear association was detected between metabolic co-dependencies and co-occurrence patterns.

**Conclusions:**

The analysis generated predictions for testable hypotheses of metabolic exchanges and co-dependencies in bacterial communities and by crossing them with co-occurrence profiles, contextualized interaction patterns into a wider ecological perspective.

**Electronic supplementary material:**

The online version of this article (10.1186/s12864-018-4786-7) contains supplementary material, which is available to authorized users.

## Background

Metabolic interactions are one of the main factors shaping communities and ecosystems by forming complex trophic networks. In bacterial communities, metabolic exchanges are ubiquitous and play a pivotal role in determining community structure [1–8]. Bacteria also exchange metabolites with multicellular organisms, and such interactions have been a key driver of evolution, enabling eukaryotic expansion into new ecological niches and species diversification [9, 10]. Among the most studied evolutionary radiations that have depended on symbiosis are sap-feeding insects such as whiteflies, aphids, psyllids, cicadas and spittlebugs. All of them have intimate associations with maternally transmitted intracellular bacteria harbored inside specialized insect cells, termed bacteriocytes. Their main symbiotic function is to provide essential nutrients (mainly essential amino acids) enabling the dietary specialization of their hosts on phloem or xylem sap of vascular plants [11–13]. In addition, insects may harbor a diverse array of facultative, nonessential bacterial associates in the bacteriocytes or other body tissues [14]. Facultative symbionts are suggested to serve as a “horizontal gene pool”, where variation in their combinations may have functional significance [13, 15–17]. Notably, since the obligatory symbionts are exposed to an irreversible process of genome reduction that can erode their metabolic potential [18], facultative symbionts can, in some cases, complement or replace parts of the lost functions [19–21].

In recent years, metabolic approaches based on genome-driven network constructions have been applied to predict the potential metabolic dependencies and metabolic exchanges between bacterial species [3, 8, 22, 23]. Newly developed tools for genome-based metabolic reconstruction enable predicting sets of interactions formed between species combinations, and the specific exchange of fluxes within multi-species systems [24–26]. Crossing such predictions with corresponding co-occurrence patterns allows deciphering the functional significance of variations in such bacterial assemblages [4, 27]. To this end, multiple information layers are required, including symbiont co-occurrence patterns, environmental conditions, genetic background of both host and symbionts, and genome-driven predictions for symbionts’ potential activities. Here, based on the availability of both distribution patterns and bacterial genome sequences, we focused on exploring the functional significance of combinations of facultative symbionts in the sweetpotato whitefly *Bemisia tabaci* (Hemiptera: Aleyrodidae) and their potential role in shaping alternative community structures.

*Bemisia tabaci* is a major pest of several key crops worldwide [28] and is referred to as a complex of species, consisting of at least 28 morphologically indistinguishable, genetically delimited groups or species [29, 30]. All whiteflies, including *B. tabaci,* harbor the obligatory symbiont “*Candidatus* Portiera aleyrodidarum” (hereafter *Portiera*) [31], which has undergone substantial genomic reduction as other obligatory symbionts [18], but is still able to produce most of the essential amino acids [32, 33]. In addition, *B. tabaci* individuals have been reported to harbor varying combinations of one to four facultative symbionts, from the bacterial genera *Rickettsia*, *Hamiltonella*, *Wolbachia*, *Arsenophonus*, *Cardinium*, *Hemipteriphilus* and *Fritschea* [34]. The distribution patterns of facultative symbionts within the body of *B. tabaci* vary. *Arsenophonus* and *Hamiltonella*, which seem to be mutually exclusive, are strictly confined inside the bacteriocytes together with *Portiera*, along with the less frequent symbionts: *Fritschea* and *Hemipteriphilus.* In contrast, *Rickettsia*, *Wolbachia* and *Cardinium* can be seen dispersed throughout the haemolymph and other tissues, located within bacteriocytes, or both [35–37]. Overall, the presence of all symbionts (obligate or facultatives) has been recorded in the bacteriocytes.

The diverse and dynamic occurrence and co-occurrence patterns of facultative symbionts in *B. tabaci* have been proposed to be related to several aspects of the insect’s biology, including host reproduction, survival and fecundity, resistance to insecticides and capacity to transmit diseases to the host plants [34]. Although the phenotypes of most facultative symbionts have not been determined yet, *Rickettsia* for example, have been shown to positively influence various fitness measures of *B. tabaci*, including the induction of higher reproduction rate and a female-biased sex ratio [38]. The occurrence and combinations frequencies of these bacterial symbionts were investigated using a dataset of over 2000 whiteflies, representing both the largest and the most comprehensive meta-study of insects for which communities of facultative symbionts have been described [34]. In this meta-study, the two most widespread *B. tabaci* species*,* MEAM1 and MED-Q1, were found to typically harbor the facultative symbiont “*Ca.* Hamiltonella defensa” (hereafter *Hamiltonella*). A combination of *Hamiltonella* and “*Ca.* Rickettsia sp.” (hereafter *Rickettsia*) seemed to be unique to MEAM1 individuals, while combinations of *Hamiltonella* with either “*Ca.* Cardinium hertigii” or “*Ca.* Wolbachia sp.” (hereafter *Cardinium* and *Wolbachia* respectively) were unique to individuals of the MED-Q1 genetic group. Based on the physical proximity of the various bacteria within the bacteriocytes, along with the lack of correlation between specific facultative symbiont complexes and any of the environmental factors tested [34], we hypothesized that metabolic interactions may be involved in shaping the bacterial community structure. The release of the genome sequences of *Portiera*, *Rickettsia*, *Hamiltonella*, and *Cardinium* [21, 39–43] has promoted the analyses of the interactions between the obligatory symbiont *Portiera* and its *B. tabaci* host, suggested to be required for the completion of essential metabolic pathways. Branched Chain Amino Acids (BCAs), for example, are synthesized through *Portiera*–host complementary interaction [41–43] while lysine biosynthesis can occur via *Portiera*–host or *Portiera*-*Hamiltonella* complementation [21, 42].

As metabolic cross talk is suggested to convey functional capacities associated with specific species combinations, we conducted comparative-interaction analysis considering interactions formed between pairwise combinations of co-residing symbionts. The genomes of four symbionts of *B. tabaci* were already published, and here we report the sequencing and assembly of a fifth symbiont - *Wolbachia* (Table 1). To study the potential cross talk between all pairwise combinations formed between the five symbionts we first reconstructed their respective metabolic networks and then used network analysis approaches to predict: (1) species-specific metabolic capacities in a simulated host’s bacteriocyte-like environment; (2) metabolic capacities of species’ combinations, and (3) the dependencies of each species on the different media components. Since the analysis is based solely on genomic data, it provides qualitative, generic predictions for *potential* exchanges and co-dependencies between co-residing symbiont, rather than quantitative estimates requiring transient information on the level of gene expression and/or metabolite accumulation and allowing a snapshot of metabolic fluxes in a given time point [44]. Notably, interactions differ greatly in dependence on the availability of resources [4]. Here, simulations were carried in a “bacteriocyte-like” environment that includes the set of metabolites that are predicted to be produced by the host. The metabolic activity and secretion profile of each symbiont can change the environment; however, the inclusion of resources that are produced through complementation might mask such interactions. In order to allow the simulations to delineate symbiont-symbiont interactions that can represent alternatives to host-symbiont interactions, the environment was initially limited to a minimal set of metabolites that are produced solely by the host. Subsequently, this basic environment was complemented by metabolites produced by the obligatory symbiont *Portiera* and the performances of the facultative symbionts in both environments were compared.

**Table 1:** General genomic features of the obligatory and facultative symbionts of *Bemisia tabaci* used for the metabolic analysis

Symbiont	Host species	Co-occurring symbionts	Accession	Coverage	N50 (kb)	Size (Mb)	Number of Contigs	CDS^b^	Number of ECs^c^
*Portiera*	MED*-*Q1-Spain	HC [32]	CP003835.1	41× [454, I]^a^	–	0.36	1	247	100
*Cardinium*	MED-Q1-Spain	PH [40]	GCA_000689375.1	> 600× [454, I]^a^	612	1.01	11 + 1	739	112
*Hamiltonella*	MED*-*Q1-China	P [21]	GCA_000258345.1	145 x [I]^a^	11.8	1.84	404	1806	398
*Hamiltonella*	MEAM1-USA	PR [71]	http://www.whiteflygenomics.org	NA[P, I]^a^	–	1.74	1	1695	434
*Rickettsia sp.*	MEAM1-China	PH [39]	GCA_000265225.2	138×[I]^a^	8.5	1.22	219	1397	247
*Rickettsia sp.*	MEAM1-USA	PH [71]	http://www.whiteflygenomics.org	NA[P,I]^a^	–	1.38	1	1522	264
*Wolbachia* sp.	MED*-*Q2-Israel	PARW	PRJEB15492	30×[454, I]^a^	6.3	1.25	297	1339	253

P, C, H, R, W represent *Portiera, Cardinium, Hamiltonella* (both genomes)*, Rickettsia* (both genomes) and *Wolbachia*, respectively

^a^Technology used for sequencing: 454 GS-FLX Titanium [454], Illumina [I] and PacBio [P]

^b^Number of CDS obtained using the JGI annotation pipeline

^c^Following annotation, filtering and manual curation

## Methods

### Genome assembly and annotation

Relevant genomes were collected from public databases (Table 1) with the exception of the *Wolbachia* genome that was assembled de novo. The sequence was deposited in the European Nuclear Archive (https://www.ebi.ac.uk/ena) under project number PRJEB15492. The procedure is fully described in Additional file 1. Genomes’ completeness was evaluated using CheckM [45], via its implementation on Kbase (http://www.kbase.us), also including genome of *Wolbachia* from *Drosophila melanogaster* as a reference to the assembly (Additional file 2). A standard protocol for annotation retrieval was applied for all genomes. Annotations were carried out using several genome-annotation pipelines: JGI (IMG/M) [46], Kbase (http://www.kbase.us), Rast [47] and MG-rast [47]. To estimate the accuracy and comprehensiveness of the predictions we benchmarked the EC (enzyme commission) predictions for the *Cardinium* genome, retrieved from the four pipelines, with annotations derived from a detailed manual curation. The IMG/G predictions were the most comprehensive and in highest agreement with the manual curation (Additional file 3). Hence, for consistency, annotations for all genomes were retrieved using the JGI and reciprocal BLAST searches were carried out between co-occurring symbionts in order to eliminate miss-assembled sequences. The phylogenetic origin of highly similar sequences was determined according to BLAST best hits. Finally, putative pseudogenes were predicted using GenePrimp [48]. Manual inspection was performed for all candidate pseudogenes that had an assigned metabolic function (EC number). In addition, previous annotations of *Cardinium* and *Portiera* [32, 40] were used as supportive information for pseudogene cleaning in these species. Finally, the predicted pseudogenes that had valid EC accessions were removed before conducting follow-up analyses. The number of ECs annotated functions for each genome is indicated in Table 1. The final EC lists are provided in Additional file 4.

### Metabolic activity simulations

Metabolic activity simulations were carried using the expansion algorithm [24] which allows predicting the active metabolic network (expanded) given a pre-defined set of substrates and reactions. The full expansion of the network reflects both the reaction repertoire of each species/species-combination and the primary set of compounds, termed here “source-metabolites”. Briefly, the algorithm starts with a set of one or more biochemical compounds acting as source metabolites for a feasible reaction, i.e., a reaction for which all required substrates are available. This reaction is selected out of the reaction pool and added to the network. In an iterative process, the products of the chosen reaction are turned into the new substrates, and so on. Processing of the starting-point compounds by relevant reactions increases the number of available compounds that can act as substrates for other, previously in-activated reactions. The network stops expanding when there are no more feasible reactions. Here, we described the resources available in the whitefly bacteriocyte by compiling several such published lists based on documented description of phloem content [49] and genomic-driven analyses of the whitefly genome and its interactions with symbionts [21, 32, 42, 50]. These previous genomics studies have explored in detail *B. tabaci* metabolism and yielded a description of potential nutrients that are secreted by the host as part of its interactions with its symbionts. The list is composed of metabolites produced by the host only, though each symbiont changes the environment by consuming/secreting unique set of metabolites. The limitation of the environment to host secreted metabolites allows predicting potential pairwise interactions that would otherwise be masked by alternative host-symbiont routes. These compounds were termed “source metabolites” (detailed in Additional file 5) and were used as a starting point for unfolding a network formed when considering all enzymes detected in a bacterial genomes. For each network/species we validated whether the expanded network includes a predefined list target metabolites (e.g., amino acids, nucleic acid, co-factors, Additional file 6) representing essential cellular components [25]. The production of this set of essential metabolites provided estimation for growth capacity in given environment (combinations of source metabolites). The general concept of growth simulation through the estimation of the production of target metabolites is illustrated in Additional file 7. Following running simulations in the environment that includes only substrates produced by the whitefly host, prediction analyses were also carried in an environment that was complemented by substrates produced by the obligatory symbiont – *Portiera.* The host-*Portiera* environment was produced by simulating *Portiera* activity in the host-only environment that was subsequently complemented by the new substrates The host-*Portiera* environment is detailed in Additional file 5. Simulations of the activity of the facultative symbionts were carried both in the host-only environment and in the host-*Portiera* environment.

### Prediction of complementary interactions

Complementation was predicted through a three-stage model adjusted from [23]: (1) constructing a combined set of metabolic reactions (EC accessions) for each pairwise combination; (2) simulating co-growth of symbiont pairs in the predicted environment; (3) comparing the set of metabolites produced by the combined genomes to those formed by the individual genomes. Complementary/Synergistic metabolites were those formed by species combinations but not by the individual species. No complimentary metabolites were detected for three-species combinations. A list of the complementary metabolites produced in each interaction and their mapping to KEGG pathways is provided in Additional file 8. Clustering and PCA (Principle Component Analysis) for the vectors of synergistic metabolites was carried out using R software [51] and modified with Inkscape.

### Prediction of co-dependencies in source metabolites

The competition scores for each pair of symbionts were calculated by the network-based tool NetCmpt that estimates the effective metabolic overlap between bacterial pairwise combinations [25]. Briefly, the tool takes as input the EC content of bacterial species, translates enzymatic content into species-specific topological networks and applies a topology-based algorithm for the prediction of species-specific metabolic resources [52]. Growth simulations (production of target metabolites) are carried for each bacterial pair member in an “optimal” environment – where all predicted resource metabolites are available, vs. reduced environment where source metabolites that are common for both pair members are excluded. The excluded shared source metabolites are assumed to represent these resources the bacteria compete over. The competition scores for each pair member are calculated by comparing the number of the produced target metabolites (Additional file 6) in the reduced environment to these formed in the optimal, non-reduced environment. The score provides a quantitative approximation to the level of effective metabolic overlap in a generic environment assuming all relevant resources are available [25]. The procedure is illustrated in Additional file 7.

Beyond the quantitative estimates, NetCmpt was further extended to identify dependencies on specific source metabolites within the relevant environment. To this end, simulations of production of target metabolites were carried in the bacteriocyte-like environment used throughout the analysis, rather than in the optimal environment used for the generic NetCmpt calculations. Within each simulation, a single metabolite was reduced from the reference bacteriocyte-like environment and the number of essential metabolites that could not be produced following the removal of the specific source metabolite was recorded.

## Results

### Assembly of the *Wolbachia* genome and metabolic reconstructions

The *Wolbachia* endosymbiont of *B. tabaci* (strain wBt-MED) was assembled in 297 contigs showing an average coverage of 10X and 20X for the Illumina and 454 libraries respectively (Table 1). To assess the quality of the assembly, first we used CheckM obtaining a 97% completeness value (Additional file 2). As a second step, the genomic features and the inferred proteome of *Wolbachia* of wBt-MED were compared against four *Wolbachia* strains from different insects (Additional files 1 and 9). Assignment of proteins into clusters of orthologues indicates that most of the proteins from *Wolbachia* of wBt-MED were present in the other *Wolbachia* strains (73%). Proteins unique to the *Wolbachia* from *B. tabaci* (27%) were mainly composed of mobile elements and proteins with unknown functions (including ankyrin repeat containing proteins). Finally, only 19 of the unique proteins were assigned with a metabolic function, yet all functions were already present in the other analyzed *Wolbachia*.

Together with the newly assembled *Wolbachia* genome, the complete genomes of *Portiera*, *Cardinium*, *Hamiltonella* and *Rickettsia* from *B. tabaci* species were retrieved from public resources (Table 1) and annotated as described in Methods section. For *Portiera*, four genomes were available [32, 33, 53] and their gene contents were compared. All strains possess a nearly identical enzymatic content (Additional file 10) and detected differences are due to errors in some of the original assemblies caused by the genome instability shown by this symbiont [33, 41]. For both *Hamiltonella* and *Rickettsia,* two genomes were available. *Rickettsia* genomes were derived from two *B. tabaci* MEAM1 populations co-harboring *Portiera* and *Hamiltonella*. *Hamiltonella* genomes were derived from *B. tabaci* MEAM1 and MED species co-harboring *Portiera* and *Rickettsia* and *Portiera* only, respectively (Table 1).

Since differences were detected in the enzyme content between the assemblies (Additional. file 11) both genome releases of *Rickettsia* and *Hamilonella* were analyzed (Table 1). For each bacterium, a metabolic-network was reconstructed based on the identification of its genome-derived enzyme content.

### Metabolic capacities of individual symbionts in the simulated bacteriocyte environment

Given a representation of data as a network, computational simulations allow addressing the influence of environmental inputs (nutritional resources) on its structure and composition, i.e., the metabolic capacities of a species in a given environment, for example, in terms of its ability to produce essential metabolites. More specifically, expansion algorithms generate the set of all possible metabolites that can be produced given a set of starting compounds (source-metabolites) and a set of feasible reactions [24]. We defined the starting compounds as a compilation of putative nutrients provided by the host whitefly in the bacteriocyte environment [21, 41, 42, 50]. Our predicted bacteriocyte environment was composed of 49 compounds including ATP, co-factors and vitamins such as NAD+, heme and thiamine, six non-essential amino acids, and sugars (Additional file 5).

**Fig. 1:**
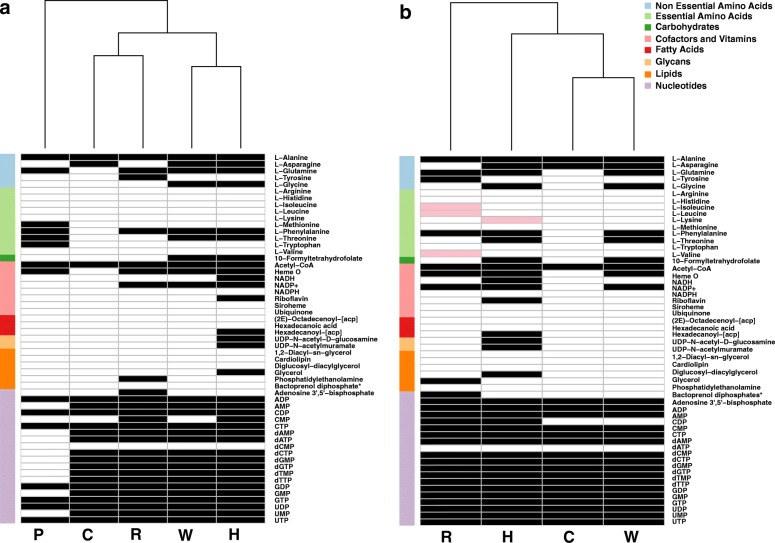
Predictions for the production of essential metabolites by symbionts in the bacteriocyte in an environment simulating substrates produced by the host (**a**) and in an environment simulating substrates produced by the host and its obligatory symbiont *Portiera* (**b**). Only essential metabolites that are not directly provided in the environment are shown. Cell color: white/black/pink – no synthesis /synthesis/synthesis unique to host-*Portiera* environment (**b**). Color on the left side indicates the super-pathway classification of each metabolite. P, C, H, R and W represent *Portiera, Cardinium, Hamiltonella, Rickettsia* and *Wolbachia*, respectively. As identical profiles were retrieved for the two *Rickettsia* and two *Hamiltonella* genomes, for simplicity only a single representative is shown

For each of the symbionts we simulated the metabolic activity in the bacteriocyte environment and listed a sub-set of essential metabolites predicted to be produced (Additional file 6). Under these conditions, most of the facultative symbionts are predicted to be capable of producing nucleic acids whereas their ability to produce amino acids and co-factors varies (Fig. 1a). *Portiera*, being an obligatory symbiont that has undergone substantial genomic reduction, was the most limited in its metabolic capacities. It was capable of synthesizing glutamine and alanine and the essential amino acids threonine, methionine (from homocysteine), tryptophan and phenylalanine, in accordance with previous reports regarding its metabolic capacities and interactions with the whitefly host [21, 32, 33, 41, 42, 50]. In addition, asparagine could be produced by the facultative symbionts *Hamiltonella, Wolbachia* and *Cardinium* and glycine by *Hamiltonella* and *Wolbachia*. Overall, the automatic-based predictions for metabolic capacities of the symbionts in the host environment generated by the model were in general agreement with previously reported genome analyses.

### Complementary interactions between the obligatory and facultative symbionts

Simulations were initially carried in an environment considering host contribution only (Fig. 1a). Since *Portiera* is the obligatory symbiont and could be considered an organelle-like entity [41], we repeated simulations in an environment that considers both host and *Portiera*’s contributions. Metabolites produced by *Portiera* through simulations in the host-only environment were added to the original environment. Simulations then compared the metabolic capacities of the facultative symbionts (all can be found in the bacteriocyte) in both environments (host vs. host-*Portiera*), showing an overall similarity (Fig. 1b). Metabolites whose synthesis depends on outputs derived from *Portiera* activity include lysine production by *Portiera-Hamiltonella* combination, in agreement with previous reports [21, 42], and production of the three BCAs (leucine, valine and isoleucine) by the *Portiera-Rickettsia* combination (Fig. 1b). This previously unreported complementation of BCA synthesis is in agreement with identification of the *ilvE* (Branched-chain-amino-acid aminotransferase) gene in *Rickettsia* from *B. tabaci*, carrying the final reaction in the BCA-synthesis pathway [54]. Co-production of amino acids by interactions of *Portiera* with the insect host were previously reported, allowing the whitefly to complement the majority of its amino-acids production [21, 32, 33, 41, 42, 44, 50]. Interactions between the obligatory and facultative symbionts can provide an alternative synthesis route, as illustrated in Additional file 11.

### Complementary interactions between the facultative symbionts

**Fig. 2:**
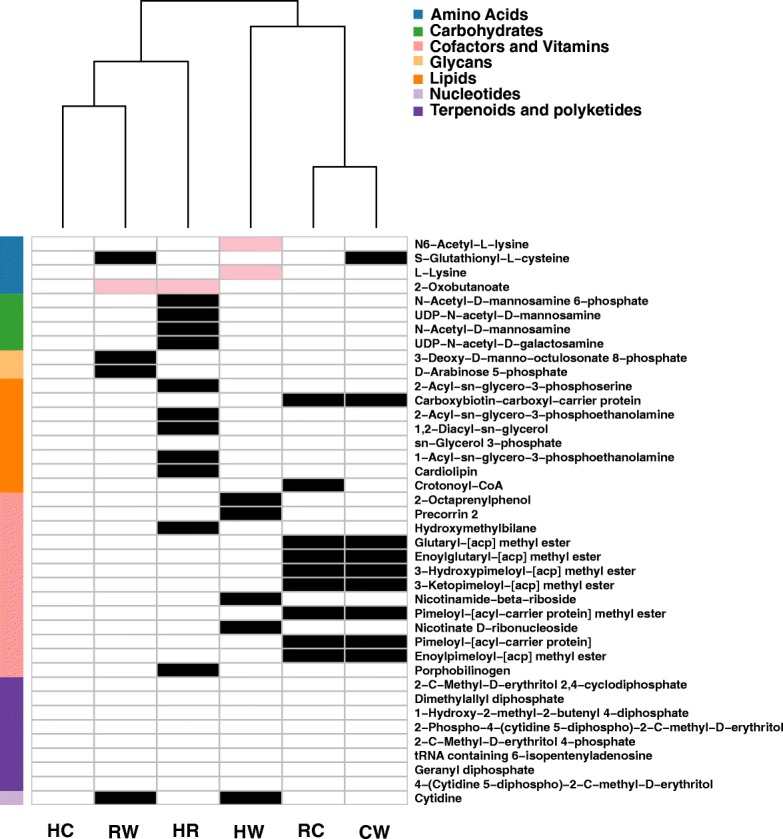
Potential ability of pairwise combinations of facultative stmbionts to synthesize complementary metabolites in the predicted bacteriocyte environment (host and host-*Portiera*). Complementary metabolites are those whose synthesis required the coexistence of both pair members and cannot be produced by either member alone in the predefined environment in which the simulations were carried out. White/black/pink coloring of the cells –no synthesis/synthesis in both environments/synthesis only in the host environment, respectively. Color on the left side indicates the super-pathway classification of each metabolite. C, H, R, W represent *Cardinium, Hamiltonella* (MEAM1)*, Rickettsia* (MEAM1-USA) and *Wolbachia*, respectively. For simplicity, only a single representative of each genome and only metabolites classified to metabolic pathways are shown; the full list of potential complementary metabolites is detailed in Additional file 8

We next looked at the potential complementary interactions between the facultative symbiont residents of the whitefly bacteriocyte. To predict potential complementation patterns, we repeated co-growth simulations for *pairwise combinations* in the exact same environments (host only and host-*Portiera*) as for single-species simulations. A metabolite was defined as “complementary’” if its synthesis requires a combination of the metabolic networks of two facultative symbionts (i.e.*,* cannot be produced by individual members of the combination). Complementary metabolites that are predicted in host-only environment and not in host-*Portiera* environment (Fig. 1b), are such that are masked by interactions of *Portiera* and the facultative symbionts – that is, can also be produced by interactions between the facultative symbiont and *Portiera*. An example for the effect of the environment is demonstrated in Fig. 2, that for simplicity focuses on subset of combinations (a single representative for each genome) and a subset of complementary metabolites (such that are mapped to a metabolic pathway). Only few of the complementary metabolites produced by interaction between the facultative symbionts are redundant with *Portiera*-facultative symbiont interactions (Fig. 2, Table 2). An example is the complementary production of lysine by a combination of *Hamiltonella* with *Wolbachia*, which is masked in the host-*Portiera* environment as it can be produced by *Hamiltonella* and *Portiera* (Fig. 2, Additional file 11).

**Table 2:** Predictions of pairwise interactions in the bacteriocyte system between occurring (bold) and non-occurring (underlined) pairwise combinations of symbionts.

	*Hamiltonella* MED-Q1	*Hamiltonella* MEAM1	*Rickettsia* MEAM1-USA	*Rickettsia* MEAM1-China	*Cardinium*	*Wolbachia*	***Portiera***
*Hamiltonella* MED-Q1		0/0 (0.32)	22/17 (0.19)	21/20 (0.2)	**0/0 (0.08)**	**11/14 (0.22)**	**13 (0.06)**
*Hamiltonella* MEAM1	0/0 (0.97)		**19/20 (0.17)**	**20/21 (0.19)**	0/0 (0.05)	18/7 (0.19)	**14 (0.05)**
*Rickettsia* MEAM1-USA	22/17 (0.2)	**19/20 (0.2)**		0/0 (0.95)	9/9 (0.09)	**6/4 (0.11)**	**14 (0.07)**
*Rickettsia* MEAM1-China	21/20 (0.21)	**20/21 (0.21)**	0/0 (1)		10/11 (0.12)	**8/7 (0.14)**	**13 (0.07)**
*Cardinium*	**0/0 (0.17)**	0/0 (0.15)	9/9 (0.1)	10/11 (0.12)		9/9 (0.12)	**13 (0.15)**
*Wolbachia*	**11/14 (0.38)**	18/7 (0.36)	6/4 (0.3)	87 (0.34)	9/9 (0.14)		**8 (0.12)**
***Portiera***	**13 (0.25)**	**14 (0.25)**	**14 (0.25)**	**13 (0.25)**	**13 (0.25)**	**8 (0.25)**	

Occurrence versus non-occurrence was determined according to a detailed survey of symbiont occurrence from 2030 whitefly individuals [34] and is detailed in Additional file 12. The first values in each cell represent the number of complementary metabolites produced in each combination in the host environment/host-*Portiera* environment (for the combinations of facultative symbionts); the value in parentheses represents the predictions of the competition values (Effective Metabolic Overlap). The obligatory symbiont is denoted in bold face

A reverse environmental effect - that is complementary metabolites that are unique to the host-*Portiera* environment were not detected, suggesting that three species complementary interactions (*Portiera* and two facultative symbionts) are rare. Overall, most metabolites produced by obligatory-facultative combinations differ from these formed by facultative-facultative interactions and the profiles derived from facultative-facultative interactions are also typically divergent and unique for each combination (Fig. 2).

**Fig. 3:**
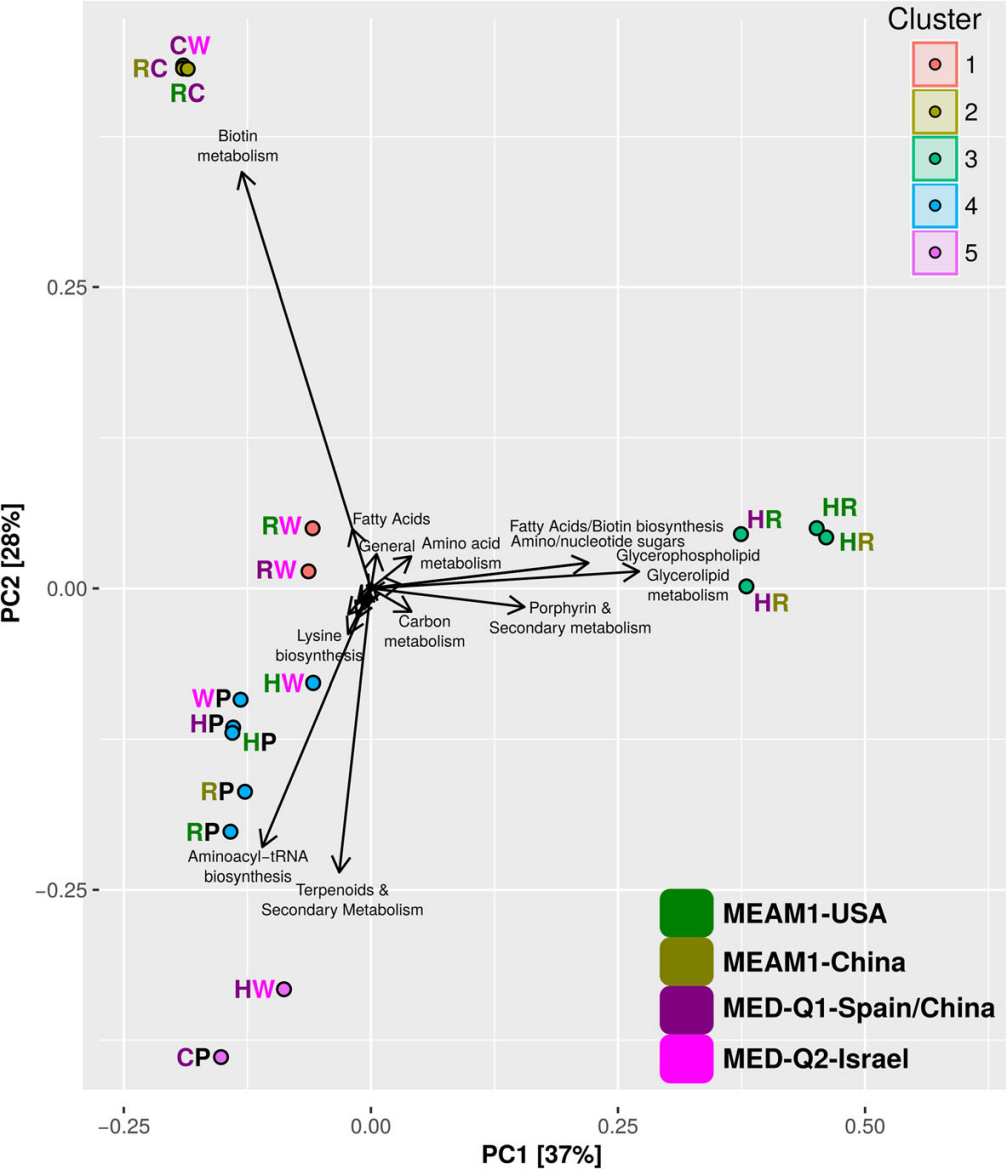
Principal Component Analysis (PCA) diagram of the complementary metabolite profiles produced through pairwise interactions (host environment). Complementary metabolites are those whose synthesis required the coexistence of both pair members and cannot be produced by either member alone in the predefined environment in which the simulations were carried out. P, C, H, R and W represent *Portiera, Cardinium, Hamiltonella, Rickettsia* and *Wolbachia*, respectively. Colors denote the species/biotype origin of each symbiont with the exception of *Portiera* that represents a generic origin. PCA clusters are present in colored circles adjacent to the bacterial combination initials. *Hamiltonella* and *Cardinium* combination have no synergistic metabolites and consequently is not represented. Black arrows represent determinant vectors. For plotting reasons, only names of the most important vectors are displayed (description of all synergistic metabolite and pathways is described in Additional file 8)

PCA of the full complementary-metabolite vectors, considering all complementary metabolites and all combinations, also points at a conservative profile of interactions between *Portiera* and the facultative symbionts versus diverse interaction profiles between the facultative symbionts (Fig. 3). *Portiera* associated interactions (with *Hamiltonella*, *Rickettsia* and *Wolbachia*, cluster 4) can potentially lead to the synthesis of a set of metabolites that includes aminoacyl-tRNAs and many primary metabolites such as amino acids and co-factors (Additional file 8). Complementary metabolites common to the co-clustered *Portiera-Hamiltonella* and *Portiera-Wolbachia* combinations included potential precursors of methionine and purine/thiamine; all potential interactions have been previously suggested for *Hamiltonella* [42], but not for *Wolbachia*. Other interaction clusters, mainly combinations of facultative symbionts, include combinations between *Cardinium*-*Portiera* (not classified together with the other *Portiera* associated combinations) and *Hamiltonella*-*Wolbachia* (cluster 5), *Hamiltonella*-*Rickettsia* interactions (cluster 3), *Rickettsia-Wolbachia* interactions (cluster 1)*,* and *Cardinium*-*Wolbachia* and *Cardinium-Rickettsia* (cluster 2). The two *Rickettsia* reconstructions lead to similar complementation profiles, unlike the significant differences that were detected between the two *Hamiltonella* reconstructions, in particular regarding to their complementation patterns with *Wolbachia* (Fig. 3). Whereas clusters 5 and 3 represent combinations that are frequently detected, most combinations in clusters 1 and 2 are less frequent (Additional file 12). The highest numbers of complementary metabolites was recorded for the frequent *Hamiltonella*-*Rickettsia* combinations (cluster 3, ~ 20 with small variations between genomic versions, Table 2) followed by *Hamiltonella*-*Wolbachia* in dependence with the specific *Hamiltonella* genome (cluster 5 & 1, Table 2). The lowest number of complementary metabolites over pairwise interactions was predicted for *Cardinium* (average of ~ 6, Table 2), the symbiont with the lowest total number of appearances in the surveyed populations. The overall pattern remains conserved in the two environments considered (Table 2). The relatively high number of complementary metabolites and the divergent clustering pattern recorded for the combinations in clusters 5 and 3 is mostly related to the co-production of secondary metabolites, fatty acids and glycerolipids (Additional file 8). Notably, combinations from clusters 5 and 3 are associated with distinct genetic backgrounds of the whitefly host. *Hamiltonella* with *Rickettsia* (cluster 3) are typical and mostly unique of individuals from MEAM1, whereas combinations of *Hamiltonella*-*Wolbachia* and *Cardinium*-*Portiera* are characteristic of individuals from MED-Q1 [34].

### Co-dependencies of symbionts on specific media components

Under the assumption that highly similar metabolic demands may hint at resource competition and potentially lead to exclusion of the less fit competitor, the extent to which symbiont combinations rely on common resources was assessed. Scores were evaluated using NetCmpt, which provides predictions for the degree of effective metabolic overlap between pairs of bacterial species, ranging between 0 (no overlap) and 1 (complete overlap) [25]. Scores are a-symmetrical whereas the effect of interactions on pair members is likely to differ (i.e.*,* one of the species is likely to be more affected than its potential competitor). The score is indicative of the effect of the column species over the row species. For example, *Hamiltonella* was almost unaffected by *Portiera* and was more sensitive to the presence of *Wolbachia* and *Rickettsia* (Table 2). Overall, pairwise scores were relatively low, ranging between 0.05–0.06 (the effect of *Portiera* on the two *Hamiltonell*as) and ~ 0.38–0.36 (the effect of the two *Hamiltonella* on *Wolbachia*). The observed average competition score, 0.18 (Table 2), was relatively low compared to an average of 0.36 calculated for other modeled bacterial communities [3]. Notably, no significant difference was observed in the level of metabolic overlap between occurring versus non-occurring combinations (t-test: t_df = 23.48_ = 0.57, *p* = 0.56; Table 2).

**Fig. 4:**
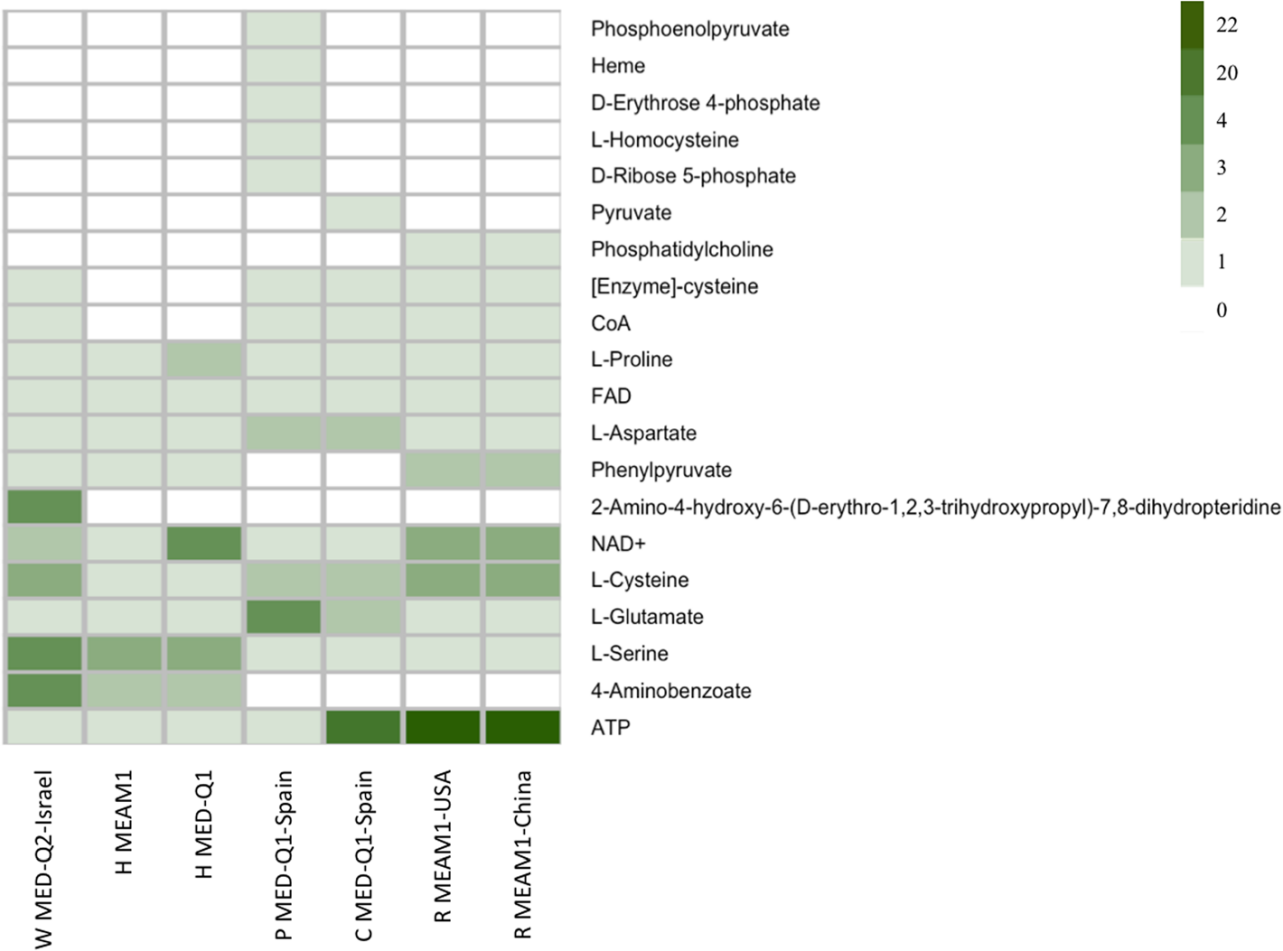
Reduction in symbiont’s ability to produce essential metabolites following removal of specific source metabolites (metabolites predicted to be available to the symbionts in the bacteriocyte) from the simulated host-environment. Only source metabolites whose removal affected at least one species are shown. P, C, H, R and W represent *Portiera, Cardinium, Hamiltonella, Rickettsia* and *Wolbachia*, respectively. Square strength color (white to dark green) represent the number of metabolites reduced from the network

Since resource overlap is thought to determine community structure only under limited carrying capacity of the habitat [55], we further simulated species-specific growth in the bacteriocyte-like environment (host only), rather than considering the generic optimal environment assumed by the NetCmpt tool. We estimated the specific qualitative effect of each metabolite on production of target metabolites capacity following iterative removal of one component at a time (as illustrated in Additional file 7). As expected, *Portiera* exhibited the most differentiated dependency profile of all symbionts (Fig. 4). In the specific bacteriocyte simulated environment, *Portiera* relied uniquely on D-ribose 5-phosphate, D-erythrose 4-phosphate and phosphoenolpyruvate for tryptophan production, as well as on L-homocysteine for methionine production. Metabolite dependencies that were common to more than a single symbiont included dependencies on the amino acids L-cysteine (*Wolbachia* and *Rickettsia*) and L-serine (*Hamiltonella* and *Wolbachia*). Hence, co-dependency might lead to a mutually exclusive distribution pattern, as suggested for *Wolbachia* and *Rickettsia* [34].

In addition, common dependencies on NAD+ (*Wolbachia* and *Rickettsia*) and ATP (*Cardinium* and *Rickettsia*) reflected the energy production pathways of the corresponding symbionts. All NAD+ dependent bacteria have a citrate cycle requiring NAD+ as a reducing force. *Rickettsia* and *Cardinum*, both missing glycolytic pathways, rely on the host for ATP production. Though *Rickettsia* possesses a citrate-cycle, capable of producing ATP, its activation requires thiamine diphosphate, which was not present in the bacteriocyte environment. In our simulations, *Wolbachia* was the only symbiont that could produce thiamine diphosphate from the thiamine provided through the activity of thiamine diphosphokinase. Like *Cardinum*, *Portiera* does not possess either a citrate-cycle or glycolysis pathway. However, at least to a minimal amount, ATP production can potentially occur through the activity of ATP phosphoribosyltransferase in the histidine-metabolism pathway requiring D-ribose 5-phosphate as input. In addition, *Portiera* can also obtain ATP through carotenoid biosynthesis [56].

## Discussion

We harnessed the rapidly advancing tools developed within the newly emerging field of eco-system biology to study microbial interactions in a small, closed, well-defined ecosystem. The focus on the *B. tabaci* unique bacterial community, where all species are known to reside in the bacteriocytes (though some of them can also be found in additional tissues), allowed exploring metabolic interactions between all relevant pairwise combinations, providing a detailed description of the trophic networks. Using simulation models to predict metabolic exchanges and co-dependencies we aimed at comparing metabolic capacities of symbiont combinations also considering the effect of the trophic complexity of the environments used for simulation (host only vs. host together with the obligatory symbiont).

We predicted four previously un-reported routes for transient complementary interactions for amino-acids synthesis between symbionts. These interactions can potentially increase the amount of the resulting amino acids in the bacteriocyte by providing alternative synthesis routes. Examples include complementation of the synthesis of BCAs that is possible through the interaction of obligatory symbiont (*Portiera*) with the insect host (*B. tabaci*), but also, by a previously un-reported interaction between *Portiera* and the facultative symbiont *Rickettsia*. Similarly, production of lysine was previously reported to occur through the complementary interaction between *Portiera* and *Hamiltonella* or, by *Wolbachia-Hamiltonella* interactions, as predicted here. The current analysis points at alternative production routes, possibly compensating for the limited transcriptional regulation of symbionts [57]. Such complementation can be mutualistic, increasing the total amount of essential nutritional sources for all community members. Alternatively, it might only be beneficial for specific species and reflect a parasitic life style. For example, complementary production of BCAs is possible through *Portiera*-*Rickettsia* interactions. Although an increase on the synthesis of BCA could be beneficial for the host and its symbionts, we must remark that the *Rickettsia* from *B. tabaci* is part of the *R. bellii* group which includes many pathogenic members [58, 59]*.* The complementation might reflect the dependency of *Rickettsia* on the BCA intermediates that it scavenges from the host-environment, bypassing the host’s control of BCA biosynthesis [60].

All *Portiera* interactions with the facultative symbionts were relatively conserved and involved in the production of metabolites compensating for the loss of aminoacyl-tRNAs in the *Portiera* lineage (L-tryptophanyl, N-formylmethionyl, L-methionyl and L-alanyl-tRNAs, Additional file 8) [41]. Although these losses are assumed to reflect the dependency of *Portiera* on its host [30, 31, 57], the analysis suggests alternative routes for such complementation, though it cannot provide an evolutionary justification. Unlike *Portiera* interactions, the interactions formed between *Hamiltonella* and the other facultative symbionts vary (Fig. 3), possibly reflecting adaptations that are specific to the host genomic background or environmentally related. For example, combinations of *Hamiltonella* with *Rickettsia* seem unique to individuals from MEAM1, whereas combinations of *Hamiltonella* with *Wolbachia* are commonly found in individuals from MED-Q1. Notably, both combinations, which are highly dominant in their corresponding genetic group [34], have the potential to co-produce a diverse set of primary and secondary metabolites, which can increase host fitness, favoring their maintenance on this species. Potential functional significance of the secondary metabolites produced through complementary interactions includes host-parasitoid interactions [61, 62]. For example, dimethylallyl diphosphate, a terpenoid, is involved in the metabolism of aphid’s alarm pheromones [63]; sialic acids have diverse functions in host-bacteria interactions, including as signaling molecules and nutritional sources [64] (Additional file 8).

In parallel to the prediction of potential exchanges, we also characterized metabolic co-dependencies between bacterial pairs. Co-dependencies might point at potential limiting factors that affect community structure. Predicted co-shared metabolites included the amino-acids L-cysteine (*Wolbachia* and *Rickettsia*) and L-serine (*Hamiltonell*a and *Wolbachia*). Whereas *Hamiltonell*a-*Wolbachia* combinations are frequent, *Wolbachia*-*Rickettsia* combinations are rare [34], indicating at cysteine as a potential limiting factor. Although cysteine is a non-essential amino acid that can be supplied by the host and is found in the phloem, it is the main sulfur source required for Fe-S protein biogenesis [65]. In addition, common dependencies in NAD+ and ATP, which reflect the energy-production pathways of the corresponding symbionts, can have a strong influence on symbiont co-occurrences. For example, *Rickettsia* and *Cardinium*, both missing the glycolytic pathways and relying on their host for ATP production, are not found together in the host [34]. Another example for mutual exclusive distribution pattern between facultative symbionts was observed for *Arsenophonus* and *Hamiltonella* [34]. The metabolic background for this exclusion can be similarly explored once the *Arsenophonus* genome is published.

Our analysis was based on several assumptions and limitations that should be acknowledged. First, metabolic capabilities were inferred using the sequenced genome of the species of interest. The genomes in the analysis vary in their status and quality (Table 1) including closed genomes (*Portiera, Hamiltonella* MEAM1-USA and *Rickettsia* MEAM1-USA)*,* high quality draft (*Cardinium*) and draft genomes (*Hamiltonella* MED-Q1-China, *Rickettsia* MEAM1-China and *Wolbachia*). Notably, draft genomes represent some challenges for this kind of inference, as it is impossible to know if the assembly failed to recover all the genes present in the genome. For that reason, we screened the genomes for putative pseudogenes to reduce the possibility of false positives enzymes, or missing enzymes. Despite differences in quality between the two *Hamiltonella* and two *Rickettsia* genomes, the inferred metabolic capacities derived from the different versions of their genomes were similar. However, for both *Hamiltonella* genomes, we observe differences in the complementation profile with *Wolbachia.* Although *Rickettsia* reconstructions came from the same species (MEAM1), *Hamiltonella* reconstructions are from different species (MEAM1 an MED-Q1) (Table 1). Though these differences in *Hamiltonella* reconstructions might reflect possible adaptation to the host, a detailed analysis suggests these differences are more likely to reflect the assembly quality, where predictions derived from the closed genome version seems more reliable and being in concordance with previous studies reporting conservation in metabolic activity between the *Hamiltonella* two strains [42]. For that reason, in the de novo *Wolbahia* assembly reported here we also screened the initial meta-assembly for missing contigs not present in the final assembly and compared the proteome of the *Wolbachia* from *B. tabaci* to the proteome of *Wolbachia* from different hosts (Additional file 9). In general, and similar to other *Wolbachia* strains sequenced, *Wolbachia* from *B. tabaci* contains a small set of real strain specific genes (13% of its proteome after some manual checks), without any strain specific enzyme, and share most of its gene content with other *Wolbachia* [66]. In summary, although we cannot rule out the possibility of missing genes in our *Wolbachi*a from *B. tabaci*, the assembly and the metabolic inference seems quite reliable for the purpose of this work.

Additional assumptions include the following: (1) we assumed a free flux of metabolites between the host and the symbionts and among the symbionts themselves. Several descriptions of the frequent exchanges in microbial communities support this assumption [3, 26, 44, 67]. (2) The model is qualitative, only providing binary predictions for the production or absence of a metabolite rather than quantitative estimates for metabolite consumption/production as produced for stoichiometric networks using constraint based modeling. Hence, metabolites that are common resources for several symbionts might not induce competition, as they are not necessarily limiting. Similarly, the coproduction of nutrients might take place in negligible amounts. The importance of quantitative analysis for deciphering host-symbiont and symbiont-symbiont interactions was demonstrated in a recent study exploring the interactions between the whitefly host, *Portiera* and *Hamiltonella* [44]. Based on quantitative estimates of co-exchange metabolic fluxes, Ankrah et al. [44] characterized *Portiera* but not *Hamiltonella* as a key source for essential amino acids for the host and suggested that *Hamiltonella* is a nutritional parasite, competing with *Portiera* for resources provided by the host. Admittedly, such interpretation cannot be obtained based on the qualitative analysis done here. Yet, in the absence of detailed metabolomics and fluxes information, any quantitative analysis has to rely on a series of assumptions and approximations. Notably, species interactions are far from being static and changes in metabolic content induce significant changes in interaction types – from competition to cooperation [4, 5, 68]. Quantitative approaches indicate the myriad of potential interactions based on genomic potential and hence are complementary to qualitative. (3) The analysis is limited to the prediction of metabolic interactions between symbionts; non metabolic interactions and host-symbiont interactions are likely to be central factors affecting community structure. However, despite these limitations, the analysis successfully captured previous genome-based predictions of metabolic complementations in the bacteriocyte [21, 32, 42]. Such evidence supports the relevance system-level genomic analyses as a tool for the formulation of new and testable predictions of metabolic exchanges in an automated manner. Moreover, our simulations take into account specific environments, hence reflecting the common notion that interactions are dynamic and can vary with the addition or depletion of nutrients [3, 5, 54]. Despite its obvious limitations, this model provides a standard account of the metabolic capacities of all symbionts as individuals as well as predicts possible interactions for all combinations.

## Conclusions

The analysis allows a systematic view of symbiont function and interactions and leads to the prediction of previously un-reported putative complementary interactions. In addition to predicting possible complementary routes, it allows predicting co-dependencies that were not previously considered and analyzed, hence providing a tool for generating testable hypotheses of metabolic interactions in bacterial communities. The focus on this small scale system, produces a relatively manageable number of potential interactions allowing their further experimental verification. Such task is close to impossible in most ecological environments that inhabit thousands of species, mostly un-sequenced. Though several genomic based studies have pointed at the relevance of genomic based description of pairwise interactions for understanding general assembly rules of natural communities, such studies can only point at general trends [4, 8]. The focus of the current study on this simple systems lays foundation for the systematic experimental exploration of these predictions towards elucidation their role in determining co-occurrence patterns, potentially providing a model system for more complex systems. Overall, the study presents a standard, systematic, approach for exploring interactions across pairwise combinations and contextualizing the findings in a broader ecological context by associating interactions with co-occurrence patterns and host background.

Understanding the overall metabolic interactions in a given system is of key importance in ecology and evolution and can provide a powerful tool for expanding knowledge on inter-specific bacterial interactions in various ecosystems. With respect to applied aspects, symbiotic microorganisms have been shown to influence the success rates of various biological control programs of agricultural pests [69, 70]. Attempts to establish more efficient pest-management strategies involve the removal of specific symbionts or the introduction of others, and our proposed model is expected to contribute to the efficiency and productivity of such efforts. The presented simple model system offers a level of tractability that is crucial for paving the way to the simulation, prediction and management of microbial communities that can expanded to more complex ecosystems, such as the guts of humans and livestock, water resources and soils.

## Additional files

Additional file 1: Description of symbiont’s DNA extraction and *Wolbachia* genome assembly. (DOCX 26 kb)

Additional file 2: Comparison of the completeness of the draft genomes using CheckM. (XLS 32 kb)

Additional file 3: Annotations for the *Cardinium* genome from four platforms (IMG/M, Kbase, Rast, MG-Rast) were compared with the manual annotation conducted by Santos-Garcia et al. 2014. The JGI platform had both the absolute highest number of Enzyme Commission (EC) predictions as well as the highest overlap with the manual annotation. Hence, it was selected as the standard annotation tool for all symbionts. (DOCX 113 kb)

Additional file 4: List of the Enzyme Commission (EC) numbers for endosymbionts used in the analyses. (XLS 79 kb)

Additional file 5: Predicted source metabolites in the whitefly bacteriocyte with and without consideration of the metabolic contribution of *Portiera aleyrodidarum*. (XLSX 17 kb)

Additional file 6: Additional file 6: List of essential metabolites needed for bacterial growth. (XLSX 10 kb)

Additional file 7: Illustration of the model used to simulate growth, calculate metabolic overlap, and estimate the effect of specific metabolites on metabolic production. (DOCX 179 kb)

Additional file 8: Categorization of the complementary metabolites produced in each combination of two symbionts into different metabolic pathways in the host environment with and without consideration of the metabolic contribution of *Portiera aleyrodidarum*. (XLS 55 kb)

Additional file 9: Orthologous protein clusters of five *Wolbachia* strains represented as a Euler–Venn Diagram, as described in additional file 1. (DOCX 187 kb)

Additional file 10: Comparison of annotations retrieved for the four different *Porteria* genomes using the JGI platform. (DOCX 111 kb)

Additional file 11: Illustration of the putative complementation at metabolic level detected for the synthesis of Branched Chain Amino Acids and Lysine. The *Wolbachia*’s lysine biosynthetic pathway lacks its last reaction (argD, EC 4.1.1.20), which is present in *Hamiltonella*, leading to a complementary potential production of lysine from M-DAP. Synthesis of M-DAP is consistently inferred in most sequenced *Wolbachia*, providing an intermediate compound for the biosynthesis of peptidoglycan, part of the bacterial membrane. (DOC 108 kb)

Additional file 12: Co-occurrence frequencies of facultative endosymbionts. (DOC 86 kb)

## Abbreviations

ATP: Adenosine Triphosphate
BCAs: Branched Chain Amino Acids
EC: Enzyme Commission
NAD +: Nicotinamide Adenine Dinucleotide
PCA: Principal Component Analysis
