# Supplementary material for: Modeling trophic dependencies and exchanges among insects’ bacterial symbionts in a host-simulated environment
Source: BMC Genomics. 2018 May 25;19:402. doi: 10.1186/s12864-018-4786-7 (PMC5970531; doi:10.1186/s12864-018-4786-7)
Supplement: Supplementary file 1 — Description of symbiont’s DNA extraction and Wolbachia genome assembly. (DOCX 26 kb) [file 12864_2018_4786_MOESM1_ESM.docx]

**Additional file 1: Symbiont DNA extraction and *Wolbachia* genome assembly.**

Total genomic DNA was extracted from a laboratory population of *Bemisia tabaci* MED species (Q2 biotype) maintained at Newe Ya’ar Research Center (Israel) as previously described [45][40]. Briefly, whiteflies were reared inside insect chambers with a 14-h light photoperiod on cotton plants. The cytotype of this strain was determined as AWR: *Arsenophonus* sp., *Wolbachia* sp. and *Rickettsia* sp. An enriched bacterial sample extraction was performed: whiteflies were homogenized in Krebs-Ringer buffer using a Dounce homogenizer. The homogenate was filtered through nylon membranes (1 mm, 80, 60, 20, 10 and 5 μm pore sizes), pelleted and treated with DNase to degrade host DNA (TURBO DNase, Life Technologies). Genomic DNA from the bacterium-enriched sample was extracted with JETFLEX Genomic DNA Purification Kit (Genomed) and six different genome amplifications were performed with GenomiPhi V2 (GE Healthcare). The genome amplifications were pooled and used as input for two libraries: 454 paired-end (1178 bp average insert size) and Illumina HiSeq 2000 single-end (100 bp chemistry), performed at Genoscope. 454 and Illumina raw reads were screened with FastQC v0.10 and cleaned/trimmed with prinseq [74][70]. An initial meta-assembly was performed with CLC Genomics Workbench, and contigs were classified according to BLAST best hits, GC content, read coverage and PhymmBL [75][71]. Illumina cleaned reads were digitally normalized with khmer [76][72] and mapped against the *Wolbachia* putative contigs with Bowtie2 [77][73]. Mira v4.0 (Chevreux et al., 1999)[74] was used to reassemble selected 454 and Illumina reads with the hirep_something parameter. SOAPdenovo v2.04 [78][75] was used for Illumina read reassembly (default parameters with k-mers from 31 to 63). All reassemblies were pooled, clustered (at 0.98 identity) with cd-hit [79][76] and mixed with minimus2 (AMOS package). Mixed assembly was used as the seed for an iterative mapping approach, using MIRA v4.0 and gap4 [80][77], until no more reads (11,9062 454 reads and 69,239 Illumina reads) or gaps were recovered or closed, respectively. Next, SSPACE v2.0 was used with the 454 library for scaffolding [81][78], while gap closing was performed with GapFiller v1.0 [82]. Finally, polisher with cleaned Illumina reads was used for homopolymer and putative sequencing error corrections. The final assembly had 16X coverage for the 454 library and 8X coverage for the digital normalized Illumina library.

The *Wolbachia* draft genome of *B. tabaci* (Hemiptera: Sternorrhyncha: Aleyrodidae) was annotated with the IMG/M server (JGI annotation tool) [47][43]. Pseudogenes were detected using GenePrimp [49][45[ followed by their manual inspection.

***Wolbachia* genome benchmarking**

To assess the quality of our annotation, and discard problems derived from the low coverage, a proteome comparison between four *Wolbachia* strains from different insects was performed. While the *Wolbachia* genomes from *Drosophila melanogaster* (Diptera: Brachycera: Drosophilidae) and *Cimex lectularius* (Hemiptera: Heteroptera: Cimicidae) were in a single annotated chromosome, the ones from *Diaphorina citri* (Hemiptera: Sternorrhyncha: Psyllidae) and *Dactylopius coccus* (Hemiptera: Sternorrhyncha: [Dactylopiidae](https://www.ncbi.nlm.nih.gov/Taxonomy/Browser/wwwtax.cgi?mode=Undef&id=38115&lvl=3&keep=1&srchmode=1&unlock)) were in a draft status without functional annotation (Additional file 9). For comparative purposes, the former genomes were re-annotated with Prokka v 1.12, using the default parameters for gram negative bacteria [83]. Finally, orthologous protein clusters were computed using OrthoMCL with 1.5 as inflation value and a BLAST e-value cut-off of 1e-5 [84] (Additional file 9).

Only 27% (335/1217) of the orthologous clusters of proteins are unique to *Wolbachia* of *B. tabaci*. Of these, 60% (203) are not functionally annotated and include mainly hypothetical, small open reading frames or putative pseudogenes no longer recognizable. From the 132 annotated proteins, 32 were ankyrin repeat-containing proteins and 29 were proteins associated to mobile elements (transposases or phages). Only 19 from the remaining 68 strain specific clusters had a metabolic function, or enzyme number associated with them. All of these 19 enzymes were detected in some of the other *Wolbachia* strains, pointing to some errors during the orthology search. CheckM [46] results shown that our *Wolbachia* assembly is almost complete (Additional file 2). Based on all the data presented, we considered our *Wolbachia* of *B. tabaci* assembly and annotation sufficient for the follow-up studies performed in this work.
