# Supplementary material for: Modeling trophic dependencies and exchanges among insects’ bacterial symbionts in a host-simulated environment
Source: BMC Genomics. 2018 May 25;19:402. doi: 10.1186/s12864-018-4786-7 (PMC5970531; doi:10.1186/s12864-018-4786-7)

**Additional file 3:** Annotations for the *Cardinium* genome from four platforms (IMG/M, Kbase, Rast, MG-Rast) were compared with the manual annotation conducted by Santos-Garcia et al. [40]. The JGI platform had both the absolute highest number of Enzyme Commission (EC) predictions as well as the highest overlap with the manual annotation. Hence, it was selected as the standard annotation tool for all symbionts.


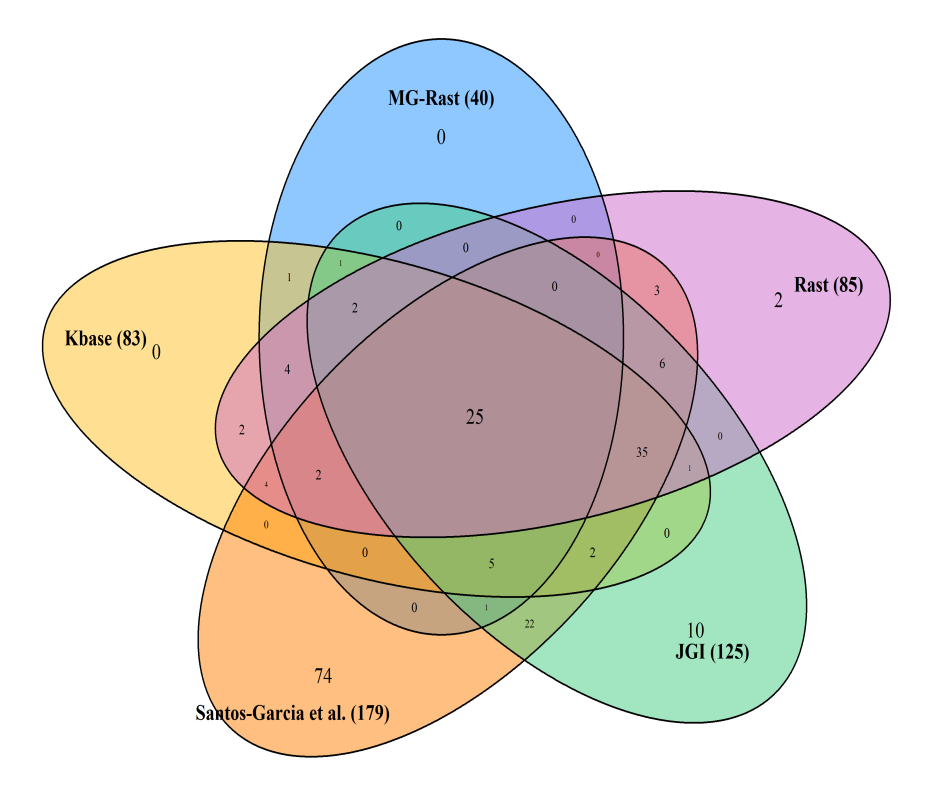

Supplement: Supplementary file 3 — Annotations for the Cardinium genome from four platforms (IMG/M, Kbase, Rast, MG-Rast) were compared with the manual annotation conducted by Santos-Garcia et al. 2014. The JGI platform had both the absolute highest number of Enzyme Commission (EC) predictions as well as the highest overlap with the manual annotation. Hence, it was selected as the standard annotation tool for all symbionts. (DOCX 113 kb) [file 12864_2018_4786_MOESM3_ESM.docx]
