# Supplementary material for: Modeling trophic dependencies and exchanges among insects’ bacterial symbionts in a host-simulated environment
Source: BMC Genomics. 2018 May 25;19:402. doi: 10.1186/s12864-018-4786-7 (PMC5970531; doi:10.1186/s12864-018-4786-7)

**Additional file** **7**: Illustration of the model used to simulate growth, calculate metabolic overlap, and estimate the effect of specific metabolites on metabolic production. EMO – Effective Metabolic Overlap (NetCmpt derived score). Red circles – source metabolites; purple circles – shared source metabolites; grey circles – metabolites in the network; green circles – target/essential metabolites; essential metabolites (green) that are not produced are circled by a grey ring; essential metabolites (green) that their production can be recovered following the addition of selected metabolite (purple) are circled by a yellow ring. Iterative simulations can be used for delineating the effect of all common metabolites (as in B) or the specific effect of selected metabolite (as in C). In the example below, grey rings are indicative of essential metabolites (green) that are not produced in the modified environment presented in panel II in comparison to the original environment in panel I. The removal of metabolite γ (panel II) leads to the loss of production of a single essential metabolite in species A (circled by the grey ring), in comparison to two other metabolites whose synthesis relies on metabolite α that is provided and are hence being produced under the modified conditions (non-ringed green circles. Similarly, in species B, the removal of metabolite β leads to the loss of three essential metabolites (circled with grey ring) vs the availability of δ preserves the synthesis of the fourth essential metabolite (non-ringed green cycle). In panel III the effect of media modification – addition of metabolites – is illustrated by comparing recovery of essential metabolites production in comparison to panel II. Addition of β allows the recovery of the synthesis of 3 essential metabolites in species B (yellow ring) and no essential metabolites in species A.


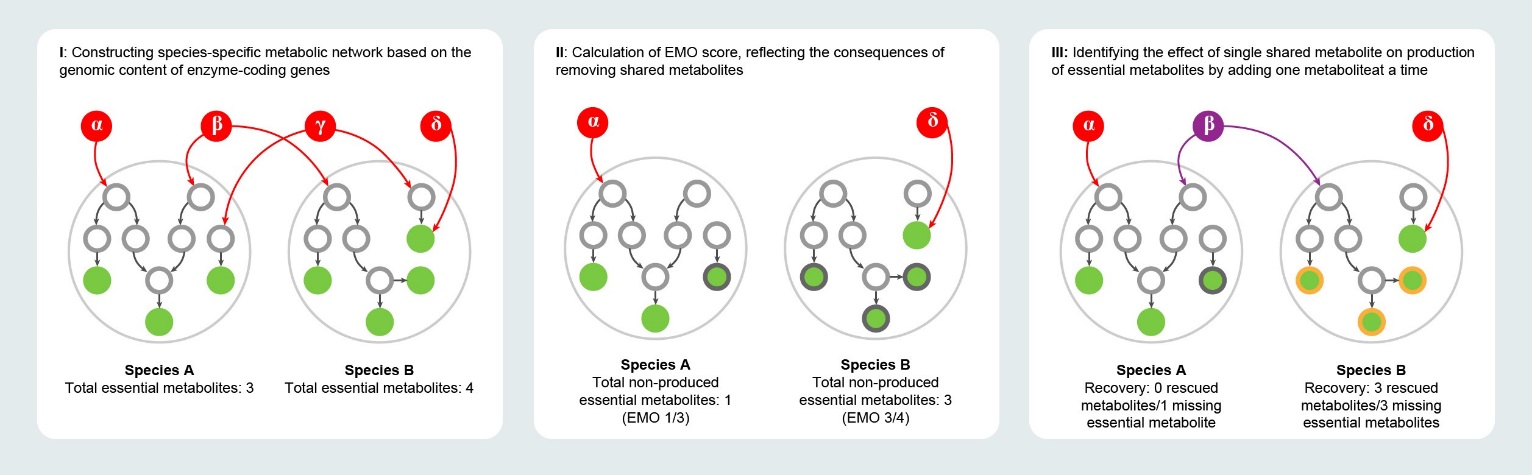

Supplement: Supplementary file 7 — Illustration of the model used to simulate growth, calculate metabolic overlap, and estimate the effect of specific metabolites on metabolic production. (DOCX 179 kb) [file 12864_2018_4786_MOESM7_ESM.docx]
