# Supplementary material for: Modeling trophic dependencies and exchanges among insects’ bacterial symbionts in a host-simulated environment
Source: BMC Genomics. 2018 May 25;19:402. doi: 10.1186/s12864-018-4786-7 (PMC5970531; doi:10.1186/s12864-018-4786-7)

**Additional file 9** Orthologous protein clusters of five *Wolbachia* strains represented as a Euler–Venn Diagram, as described in additional file 1.


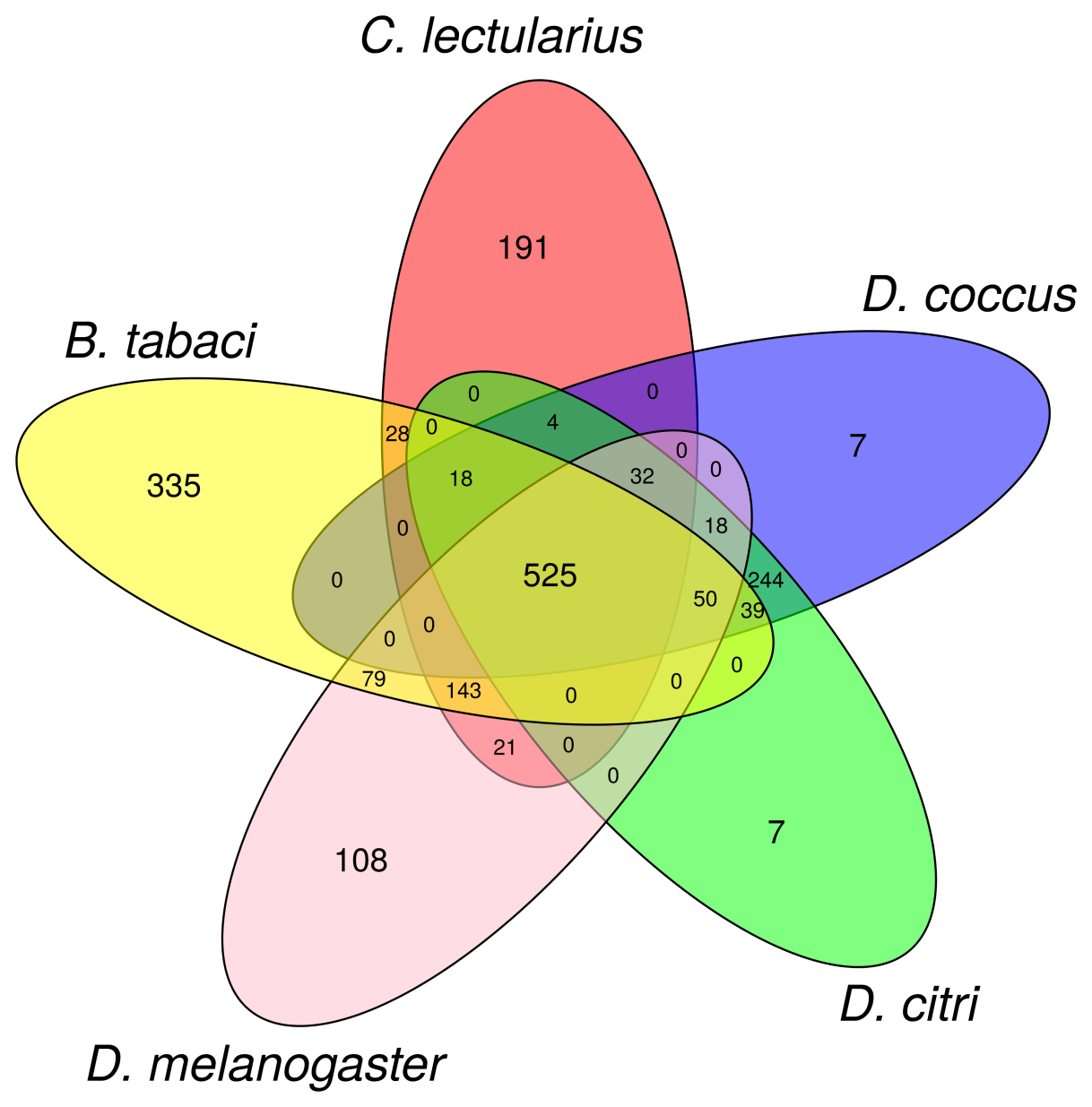

Supplement: Supplementary file 9 — Orthologous protein clusters of five Wolbachia strains represented as a Euler–Venn Diagram, as described in additional file 1. (DOCX 187 kb) [file 12864_2018_4786_MOESM9_ESM.docx]
