# Supplementary material for: Modeling trophic dependencies and exchanges among insects’ bacterial symbionts in a host-simulated environment
Source: BMC Genomics. 2018 May 25;19:402. doi: 10.1186/s12864-018-4786-7 (PMC5970531; doi:10.1186/s12864-018-4786-7)

**Additional file 10** Comparison of annotations retrieved for the four different *Porteria* genomes using the JGI platform: MEAM1 Sloan [33][32], MEAM1 Jiang [53]Jiang [47], MED-Q1 Santos-Garcia [32]Santos-Garcia [31] and MED-Q2 Jiang [53]Jiang [47]. Number in brackets indicates number of EC (Enzyme commission).


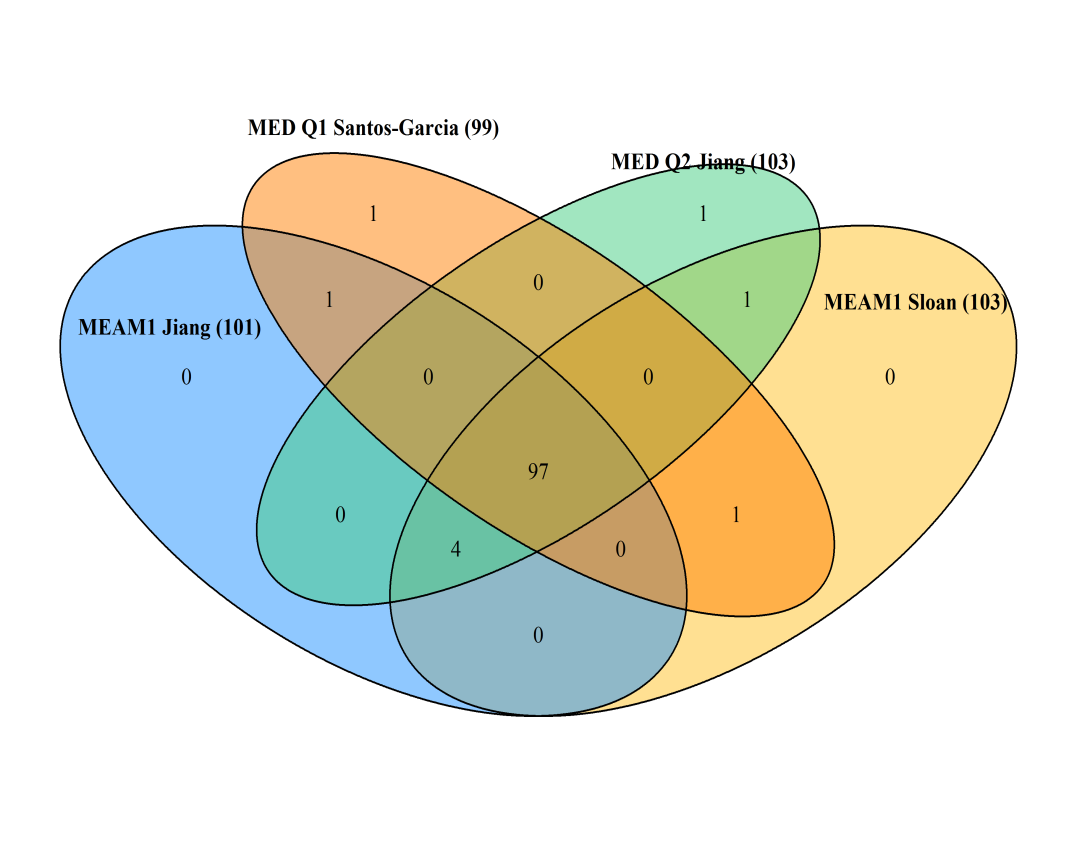

Supplement: Supplementary file 10 — Comparison of annotations retrieved for the four different Porteria genomes using the JGI platform. (DOCX 111 kb) [file 12864_2018_4786_MOESM10_ESM.docx]
