# Supplementary material for: Modeling trophic dependencies and exchanges among insects’ bacterial symbionts in a host-simulated environment
Source: BMC Genomics. 2018 May 25;19:402. doi: 10.1186/s12864-018-4786-7 (PMC5970531; doi:10.1186/s12864-018-4786-7)

**Additional file 11:** Putative complementations at metabolic level detected for the synthesis of Branched Chain Amino Acids and Lysine. As can be seen in the bottom panel, the *Wolbachia’*s lysine biosynthetic pathway lacks its last reaction (*argD,* EC 4.1.1.20), which is present in *Hamiltonella,* leading to a complementary potential production of lysine from M-DAP. Synthesis of M-DAP is consistently inferred in most sequenced *Wolbachia*, providing an intermediate compound for the biosynthesis of peptidoglycan, part of the bacterial membrane. Gray nodes represent intermediate metabolites, yellow nodes start metabolites of the pathway represented, green nodes essential amino acids. Narrow arrows represent enzymatic reactions. Broad arrows represent exchanges of metabolites between Host-*Portiera* (green), *Hamiltonella*-*Portiera* (blue), *Wolbachia*-*Hamiltonella* (red) and *Rickettsia*-*Portiera* (dark grey). Dashed arrow represent simplified pathways or the final metabolite target. M-DAP: meso-diaminopimelate.


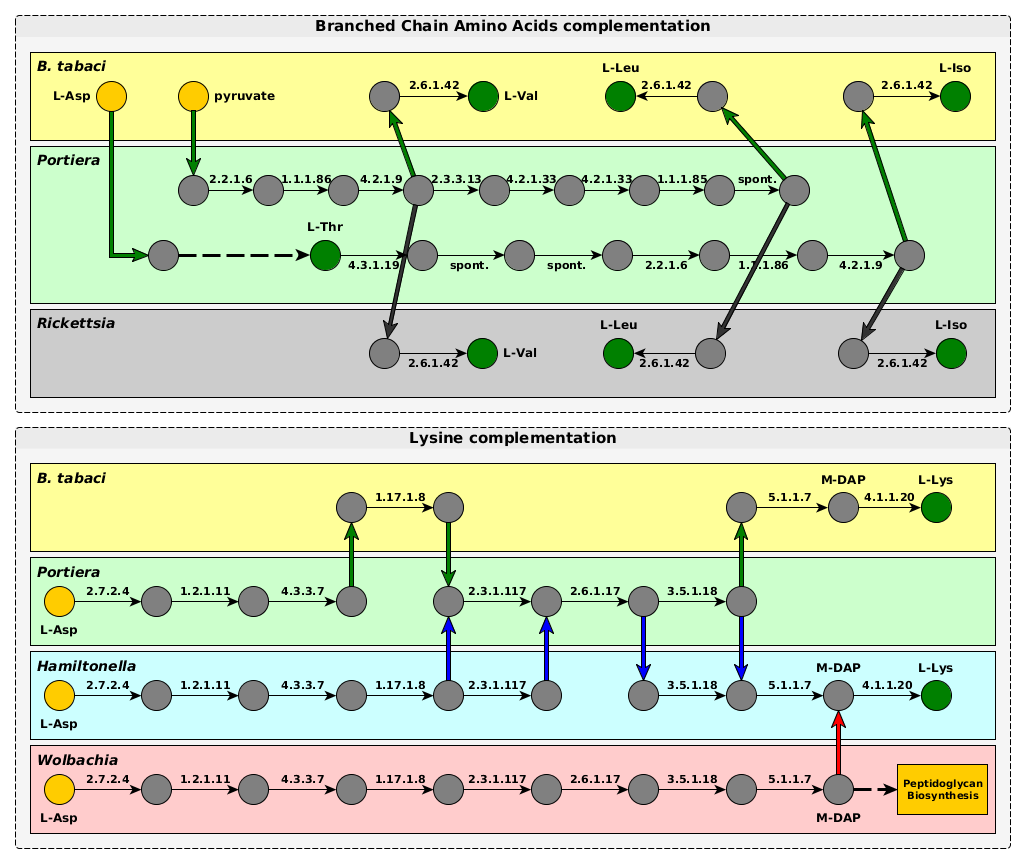

Supplement: Supplementary file 11 — Illustration of the putative complementation at metabolic level detected for the synthesis of Branched Chain Amino Acids and Lysine. The Wolbachia’s lysine biosynthetic pathway lacks its last reaction (argD, EC 4.1.1.20), which is present in Hamiltonella, leading to a complementary potential production of lysine from M-DAP. Synthesis of M-DAP is consistently inferred in most sequenced Wolbachia, providing an intermediate compound for the biosynthesis of peptidoglycan, part of the bacterial membrane. (DOC 108 kb) [file 12864_2018_4786_MOESM11_ESM.doc]
