# Supplementary material for: Modeling trophic dependencies and exchanges among insects’ bacterial symbionts in a host-simulated environment
Source: BMC Genomics. 2018 May 25;19:402. doi: 10.1186/s12864-018-4786-7 (PMC5970531; doi:10.1186/s12864-018-4786-7)
Supplement: Supplementary file 12 — Co-occurrence frequencies of facultative endosymbionts. (DOC 86 kb) [file 12864_2018_4786_MOESM12_ESM.doc]

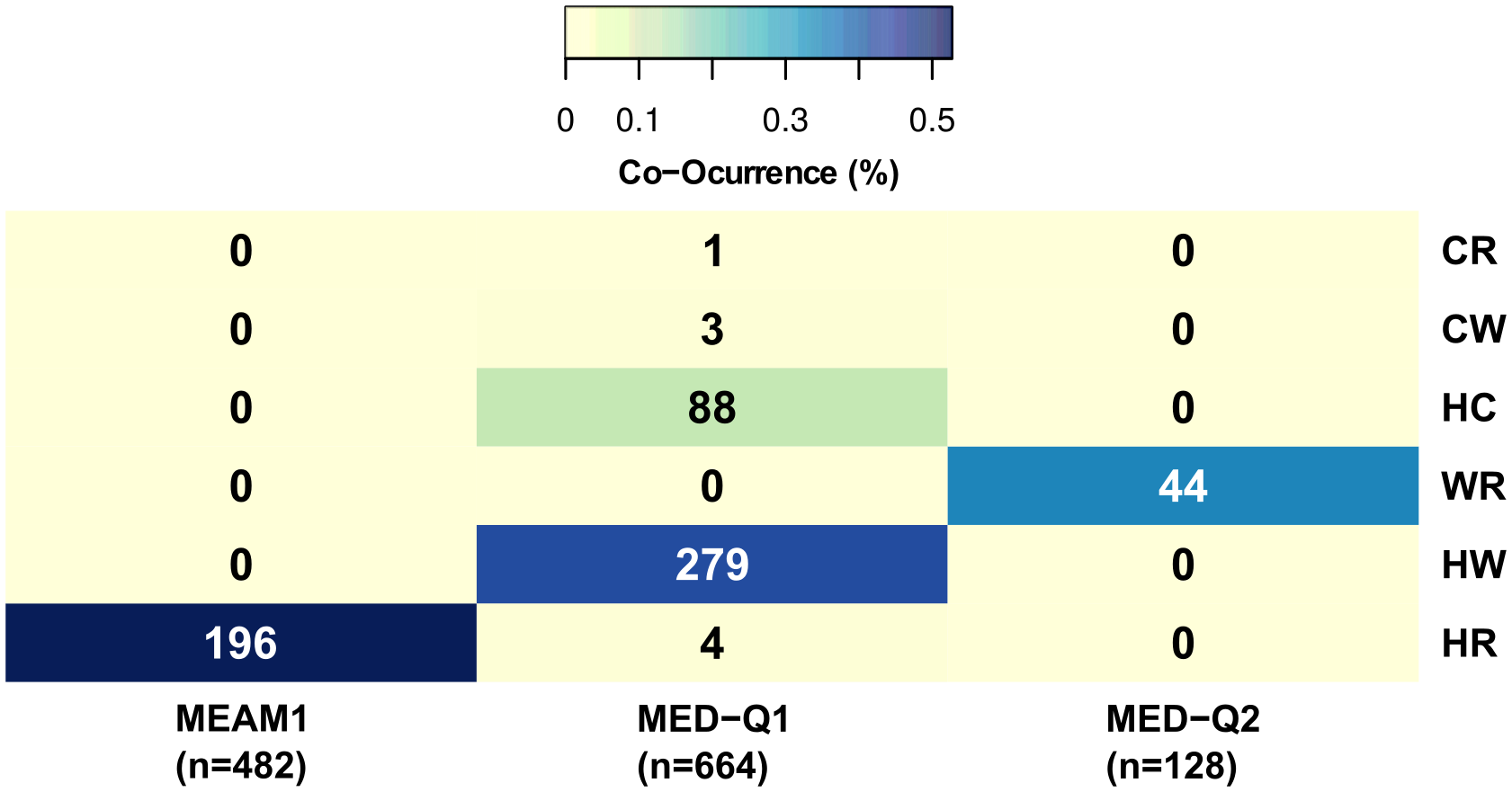
**Additional file 12:** Co-occurrence frequencies of facultative endosymbionts. Numbers inside cells represent the number of whitefly individuals in which a specific combination was detected. Total number of individuals, classified into distinct genetic groups of*B. tabaci* species is indicated for each column. Data was scaled by columns. Single symbiont occurrence was: MED-Q1 H=261, C=15, W=4, R=0; MEAM1 H=196, C=0, W=0, R=17; MED-Q2 H=0, C=0, W=10, R=53. Data was taken from Zchori-Fein *et al.* 2014 [34].
